# Increased Expression of Yes-Associated Protein 1 in Hepatocellular Carcinoma with Stemness and Combined Hepatocellular-Cholangiocarcinoma

**DOI:** 10.1371/journal.pone.0075449

**Published:** 2013-09-24

**Authors:** Gi Jeong Kim, Hyunki Kim, Young Nyun Park

**Affiliations:** 1 Department of Pathology, Yonsei University College of Medicine, Seoul, Republic of Korea; 2 Integrated Genomic Research Center for Metabolic Regulation, Yonsei University College of Medicine, Seoul, Republic of Korea; The University of Tokyo, Japan

## Abstract

Combined hepatocellular-cholangiocarcinoma (cHC-CC) and some hepatocellular carcinomas (HCCs) express stemness-related markers, such as epithelial adhesion molecule (EpCAM) and keratin 19 (K19), the expression of which has been reported to be associated with more aggressive behavior therein than in HCCs without. Yes-associated protein 1 (YAP1), a potential oncogene, is known to promote stem cell proliferation. In the present study, YAP1 expression and clinicopathological features were evaluated and compared among three groups comprising 36 HCCs that expressed both EpCAM and K19, 64 HCCs that did not express EpCAM and K19, and 58 cHC-CCs, which consisted of 38 cases of the classical type and 20 cases of the intermediate-cell subtype. YAP1 expression was more frequently noted in EpCAM(+)/K19(+) HCCs (55.6%) and in cHC-CCs (67.2%) than in EpCAM(−)/K19(−) HCCs (17.2%) (*P*<0.001 for both). In cHC-CCs, YAP1 expression was observed in 63% of classical type cHC-CCs and in 75% of the intermediate subtype; moreover, such expression was correlated with poorer histological differentiation (*P* = 0.017) and was more frequently noted in transition zones than in HCC areas (*P* = 0.060). Disease-free and overall survival showed a statistically significant difference among the three groups: disease-free survival was highest for EpCAM(−)/K19(−) HCCs and lowest for cHC-CCs, with EpCAM(+)/K19(+) HCCs falling in between (*P*<0.05). Overall survival rate was lower in HCCs and cHC-CCs with YAP1 expression compared to those without (*P* = 0.05), whereas disease-free survival showed no significant difference according to YAP1 expression. Increased YAP1 expression was more frequently found in cHC-CCs and HCCs with stemness than in HCCs without, and a YAP1 pathway is suggested to be involved in the obtainment stemness characteristics in HCCs and cHC-CCs.

## Introduction

Hepatocellular carcinoma (HCC) is the sixth most common malignancy worldwide and the third greatest cause of cancer-related mortality, especially in Asia and Africa.[1] Combined hepatocellular and cholangiocarcinoma (cHC-CC), an uncommon subtype that accounts for approximately 1% of all primary liver tumors, comprises morphologically and phenotypically mixed elements of HCC and cholangiocarcinoma (CC).[2], [3] cHC-CCs can be categorized as classical type or subtypes with stem cell features. The latter can further be subcategorized into typical subtype, intermediate-cell subtype, and cholangiocellular subtype.[3]

Recent advances in the study of cancer stem cells have indicated that cancer stem cells play a critical role in tumor growth and the progression of HCCs, contributing to their ability to self-renew, differentiate, and generate metastatic tumors in local or distant organs.[4]–[8] HCCs expressing stemness-related markers, including epithelial cell adhesion molecule (EpCAM), keratin 19 (K19), CD90, and CD133, are known to exhibit more aggressive biological behavior and worse prognosis than HCCs that express no stemness-related markers.[5], [9]–[11] As well, cHC-CCs, which also express stemness-related markers, have been reported to show more aggressive behavior than HCCs.[12], [13]

Yes-associated protein 1 (YAP1) is a major downstream target of the Hippo-signaling pathway, an evolutionarily conserved pathway from *Drosophila* to humans that is known to control organ size during development.[14]–[16] Regulation of the Hippo-signaling pathway is known to be mediated by phosphorylation and subcellular localization of YAP1. Activation of the Hippo-signaling pathway induces phosphorylation of YAP1, which prevents the translocation thereof to the nucleus. Instead, phosphorylated YAP1 remains in the cytoplasm, where it is degraded by proteasomes. When the Hippo-signaling pathway is inactivated, dephosphorylated YAP1 is translocated to the nucleus where it interacts with transcription factors, eventually leading to the proliferation of cells to various organ systems.[17]–[20] One previous study using transgenic mice with liver-specific YAP1 overexpression revealed significant increases in liver size and number of primary liver tumors morphologically resembling cHC-CC in humans.[21]

To our knowledge, the expression of YAP1 has not been investigated in primary liver cancers with stemness features. In this study, YAP1 expression patterns and clinicopathological characteristics were compared among HCCs with and without stemness-related markers and cHC-CCs.

## Materials and Methods

### Case selection and clinicopathological analysis

A total of 158 cases of primary liver carcinoma showing the following features were studied: (1) 36 HCCs expressing both EpCAM and K19 [EpCAM(+)/K19(+)], (2) 64 HCCs without expression of both EpCAM and K19 [EpCAM(−)/K19(−)], and (3) 58 cHC-CCs. The cHC-CCs included 38 cases of classical type cHC-CC and 20 cases of the intermediate-cell subtype.

Tumor specimens were fixed in 10% buffered formalin and representative sections were submitted for histological examination. The histopathological variables recorded for each case included tumor size, multiplicity, differentiation according to a three-tiered grading system (well, moderately and poorly differentiated), vascular invasion, and intrahepatic metastasis. Clinical features including age, sex, etiology, and follow-up data were obtained from hospital charts. There were 55 cases with a history of preoperative treatment including 44 cases of transcatheter arterial chemoembolization (TACE), one case of concurrent chemoradiotherapy (CCRT), five cases of TACE and CCRT, one case of chemotherapy, and four cases of radiofrequency ablation. This study was approved by the Institutional Review Board of Severance Hospital, Yonsei University College of Medicine (Seoul, Korea). The Institutional Review Board waived the need for consent in this study (4-2013-0272).

### Immunohistochemical staining

The expressions of YAP1, EpCAM, and K19 were evaluated by immunohistochemical staining in representative sections of formalin-fixed, paraffin-embedded (FFPE) tissues. Primary antibodies for YAP1 (1∶100, Cell Signaling Technology, Danvers, MA, USA), EpCAM (1∶1000, Calbiochem, Merck, Darmstadt, Germany), and K19 (1∶100, Dako, Carpinteria, CA, USA) were used. Briefly, 4-µm-thick sections of FFPE tissues were deparaffinized and rehydrated. After treatment with a 3% hydrogen peroxide solution for 20 min to block endogenous peroxidases, the sections were pretreated in 10 mM citrate buffer (pH 6.0) in a microwave oven for 20 min for antigen retrieval. After incubation with the primary antibodies, the sections were then processed using the EnVision™ Detection System (Dako) according to the manufacturer's instructions, and 3, 3′-diaminobenzidine tetrahydrochloride was used as a chromogen. All sections were counterstained with Mayer hematoxylin.

The immunoreactivities of YAP1, EpCAM, and K19 were evaluated by two independent observers (G. J. Kim and H. Kim). Conflicting cases were reviewed and discussed until a consensus was obtained. For the assessment of YAP1 expression, nuclear YAP1 expression of bile ductular epithelial cells was used as an internal positive control with moderate intensity. Non-tumor hepatocytes were used as an internal negative control. YAP1 expression was graded according to nuclear expression intensity: weak, moderate, or strong expression. Cases showing YAP1 expression in less than 5% of the tumor cells of any intensity grade or those of weak intensity were regarded as negative (no YAP1 expression), while cases showing moderate to strong intensities in more than 5% of the tumor cells were regarded as positive for YAP1 expression.

For expression of EpCAM and K19, membranous or cytoplasmic expression in more than 5% of the tumor cells was considered positive. Bile ductular epithelial cells were used as an internal positive control for EpCAM and K19. All cHC-CCs were positive for both EpCAM and K19.

### Statistical analyses

Statistical analyses were performed using SPSS software version 19.0 for Windows (SPSS Inc., Chicago, IL, USA). Fisher's exact test was used for analysis of categorical variables. Continuous variables were analyzed using one-way analysis of variance (ANOVA) or Student's t-test; these results are expressed as the mean ± standard deviation. Histological grades were compared using the Mann–Whitney U test. On survival analysis, clinicopathologic variables were dichotomized and analyzed according to their effect on prognosis. Disease-free survival and overall survival analysis was performed using the Kaplan–Meier method, and differences between the groups were assessed using the log-rank test. Univariate and multivariate survival analyses were carried out using Cox proportional hazard regression models. Only variables significant on the univariate analysis of factors affecting survival were used in the multivariate analysis. Estimated relative risks of death were expressed as adjusted hazard ratios (HR) and corresponding 95% confidence intervals (95% CI). All *P*-values less than 0.05 were regarded as statistically significant.

## Results

### Clinicopathological features of EpCAM(−)/K19(−) HCCs, EpCAM(+)/K19(+) HCCs, and cHC-CCs

The clinicopathological characteristics of EpCAM(−)/K19(−) HCCs, EpCAM(+)/K19(+) HCCs, and cHC-CCs are summarized in Table 1. Both EpCAM(+)/K19(+) HCCs and cHC-CCs developed in patients of younger age than EpCAM(−)/K19(−) HCCs (*P* = 0.001 and *P* = 0.005, respectively). Tumor size was larger in the cHC-CCs than in HCCs (cHC-CCs *vs.* EpCAM(−)/K19(−) HCCs, *P*<0.001; cHC-CCs *vs.* EpCAM(+)/K19(+) HCCs, *P* = 0.033). cHC-CCs also more frequently presented as a single lesion than EpCAM(−)/K19(−) HCCs and EpCAM(+)/K19(+) HCCs (*P* = 0.032 and *P* = 0.002, respectively). Vascular invasion was more frequently observed in cHC-CCs than in HCCs (cHC-CCs *vs.* EpCAM(−)/K19(−) HCCs, *P*<0.001; cHC-CCs *vs.* EpCAM(+)/K19(+) HCCs, *P* = 0.025). Additionally, EpCAM(+)/K19(+) HCCs and cHC-CCs exhibited poorer histological differentiation than EpCAM(−)/K19(−) HCCs (*P*<0.001 for both). Among cHC-CCs, the classical type was more frequently related to human hepatitis B virus or hepatitis C virus infection than the intermediated-cell subtype (*P* = 0.001). There was no difference between the two types in terms of sex, age, tumor size, differentiation, *et al*. (Table S1).

**Table 1:** Clinicopathological features and YAP1 expression in HCCs and combined hepatocellular-cholangiocarcinomas (cHC-CCs).

	Group 1	Group 2	Group3			
	EpCAM(−)/K19(−) HCCs (%) (*n* = 64)	EpCAM(+)/K19(+) HCCs (%) (*n* = 36)	cHC-CCs (%) (*n* = 58)	Group 1 *vs.* 2	Group 1 *vs.* 3	Group 2 *vs*. 3
**Sex**				0.892	0.810	0.943
** Male**	54 (84.4)	30 (83.3)	48 (82.8)			
** Female**	10 (15.6)	6 (16.7)	10 (17.2)			
**Age (years)**	59.6±9.7	52.4±11.8	54.4±10.5	0.001	0.005	0.378
**Etiology**				0.239	0.003	0.161
** Non-viral**	7 (10.9)	7 (19.4)	19 (32.8)			
** HBV**	50 (78.2)	28 (77.8)	35 (60.3)			
** HCV**	7 (10.9)	1 (2.8)	4 (6.9)			
**Tumor size (mm)**	33.4±16.5	38.4±21.8	50.1±30.6	0.204	<0.001	0.033
**Differentiation**				0.001	0.001	0.903
** Well**	27 (42.2)	3 (8.3)	10 (17.2)			
** Moderate**	30 (46.9)	26 (72.2)	33 (56.9)			
** Poor**	7 (10.9)	7 (19.5)	15 (25.9)			
**Vascular invasion**				0.199	<0.001	0.025
** Absence**	37 (57.8)	16 (44.4)	13 (22.4)			
** Presence**	27 (42.2)	20 (55.6)	45 (77.6)			
**Multiplicity**				0.251	0.032	0.002
** Single**	54 (84.4)	27 (75.0)	56 (96.6)			
** Multiple**	10 (15.6)	9 (25.0)	2 (3.4)			
**Intrahepatic metastasis**				0.617	0.003	0.071
** Absence**	62 (96.9)	34 (94.4)	46 (79.3)			
** Presence**	2 (3.1)	2 (5.6)	12 (20.7)			
**Preoperative treatment**				0.068	0.179	0.532
** No**	47 (73.4)	20 (55.6)	36 (62.1)			
** Yes**	17 (26.6)	16 (44.4)	22 (37.9)			
**YAP1 expression** *				<0.001	<0.001	0.255
** Negative**	53 (82.8)	16 (44.4)	19 (32.8)			
** Positive**	11 (17.2)	20 (55.6)	39 (67.2)			

HCC, hepatocellular carcinoma; cHC-CC, combined hepatocellular-cholangiocarcinoma

* Nuclear YAP1 expression with moderate to strong intensities in more than 5% of the tumor cells were regarded as positive.

### YAP1 expression in EpCAM(−)/K19(−) HCCs, EpCAM(+)/K19(+) HCCs, and cHC-CCs

**Figure 1:**
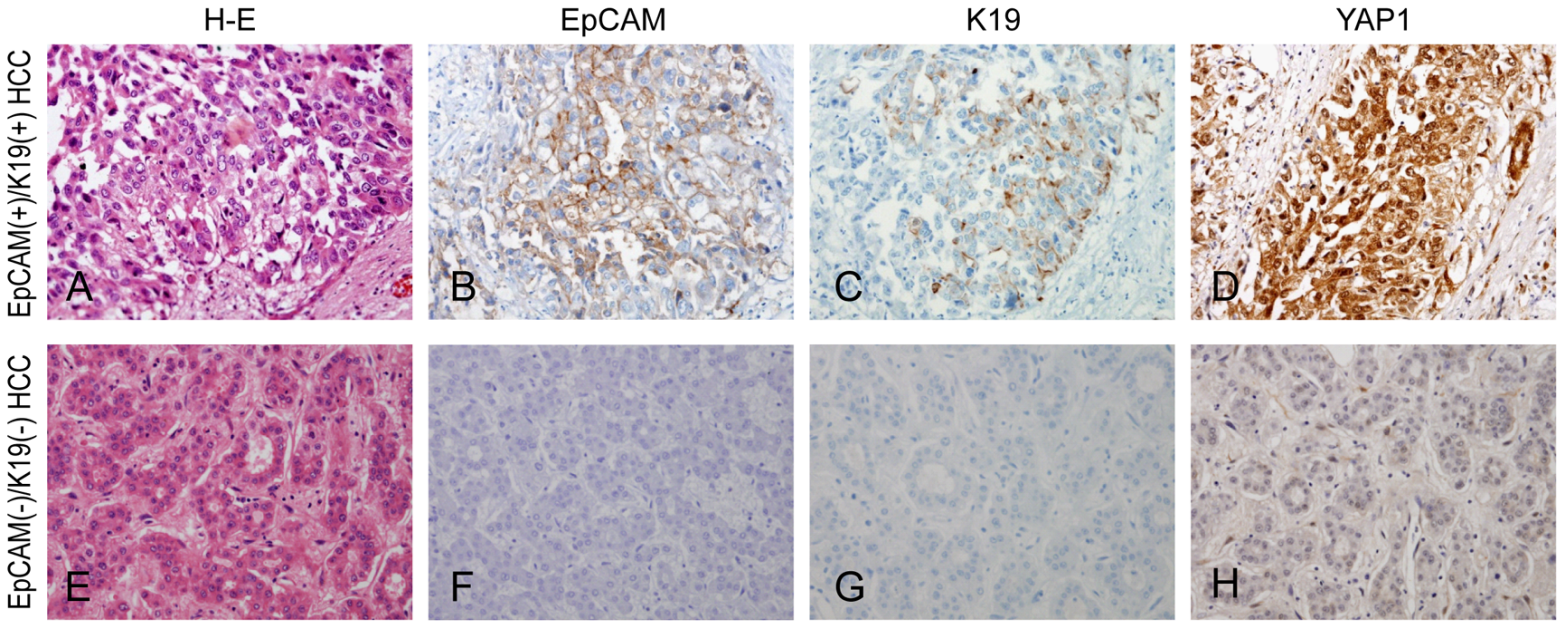
EpCAM and K19 expression in hepatocellular carcinoma (HCC). (A-D) HCC expressing both EpCAM and K19. The expression of EpCAM was mainly membranous, and K19 showed cytoplasmic expression in tumor cells. Nuclear expression of YAP1 was noted. (E-H) HCC without expression of both EpCAM and K19. There was no nuclear expression of YAP1. (Original magnification, ×200).

YAP1 expression was found in 11/64 (17.2%) EpCAM(−)/K19(−) HCCs, 20/36 (55.6%) EpCAM(+)/K19(+) HCCs, and 39/58 (67.2%) cHC-CCs (Table 1) (Figure 1). YAP1 expression was significantly, more frequently observed in EpCAM(+)/K19(+) HCCs and cHC-CCs than in EpCAM(−)/K19(−) HCCs (*P*<0.001 for both). There was no significant difference in YAP1 expression between EpCAM(+)/K19(+) HCCs and cHC-CCs.

**Figure 2:**
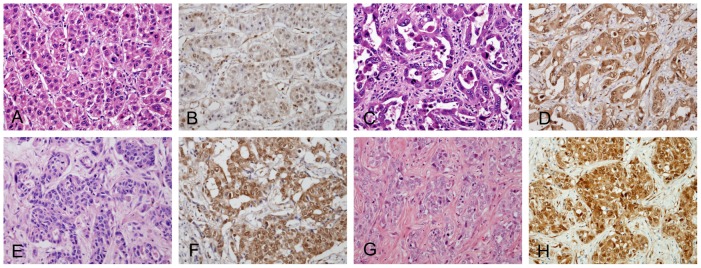
YAP1 expression in combined hepatocellular cholangiocarcinoma (cHC-CC). (A-F) Pathological features and YAP1 expression are shown in each component of classical type combined hepatocellular cholangiocarcinoma, including a hepatocellular carcinoma (HCC) area (A, B), a cholangiocarcinoma (CC) area (C, D), and a transitional zone (E, F). YAP1 expression is evident in the nuclei of tumor cells in CC areas (D) and transitional zones (F) in contrast to weak nulcear YAP1 expression in HCC areas (B). (G-H) Intermediate subtype of cHC-CC with stem cell features showing strong nuclear YAP1 expression. (Original magnification, ×200).

In cHC-CCs, YAP1 expression was present in 24/38 (63.2%) classical type cHC-CCs and in 15/20 (75.0%) intermediate subtype cHC-CCs with stem cell features, a difference that was not statistically significant. YAP1 expression was further analyzed in each histological component of classical type cHC-CCs (this analysis was performed in 29 cases of classical type cHC-CC due to a shortage of tissues). In doing so, positive expression was found in 8/29 (27.6%) HCC areas, 12/29 (41.4%) cholangiocarcinoma (CC) areas, and 15/29 (51.7%) transitional zones (Table 2) (Figure 2). YAP1 expression was more frequently recoded in transitional zones than in HCC areas (*P* = 0.060), although this was not statistically significant. The intermediate-cell subtype of cHC-CCs with stem cell features predominantly consisted of tumor cells with intermediate features between hepatocytes and cholangiocytes, which showed no zonal pattern of YAP1 expression.

**Table 2:** YAP1 expression in each histologic component of combined hepatocellular-cholangiocarcinomas (classic type).

YAP1 expression*	HCC area	CC area	Transition zone	Transition *vs.* HCC area	Transition *vs.* CC area	HCC *vs.* CC area
Positive	8 (27.6%)	12 (41.4%)	15 (51.7%)	0.060	0.430	0.269
Negative	21 (72.4%)	17 (58.6%)	14 (48.3%)			

HCC, hepatocellular carcinoma; CC, cholangiocarcinoma

* Nuclear YAP1 expression with moderate to strong intensities in more than 5% of the tumor cells were regarded as positive.

The clinicopathological characteristics of HCCs and cHC-CCs according to YAP1 expression are summarized in Table 3. Among EpCAM(+)/K19(+) HCCs, cases with YAP1 expression more frequently manifested as a single lesion than those that did not express YAP1 (*P* = 0.005). Among cHC-CCs, the expression of YAP1 was associated with poorer differentiation (*P* = 0.017), whereas both EpCAM(−)/K19(−) HCCs and EpCAM(+)/K19(+) HCCs showed no difference in tumor differentiation according to YAP1 expression. There were no differences in tumor size and vascular invasion according to YAP1 expression for all groups.

**Table 3:** Clinicopathological characteristics of HCCs and combined hepatocellular-cholangiocarcinomas according to YAP1 expression.

	EpCAM(−)/K19(−) HCCs			EpCAM(+)/K19(+) HCCs			cHC-CCs		
	YAP1 negative (%) (*n* = 53)	YAP1 positive (%) (*n* = 11)	*P*	YAP1 negative (%) (*n* = 16)	YAP1 positive (%) (*n* = 20)	*P*	YAP1 negative (%) (*n* = 19)	YAP1 positive (%) (*n* = 39)	*P*
**Sex**			0.356			0.196			0.142
** Male**	46 (86.8)	8 (72.7)		15 (93.8)	15 (75.0)		18 (94.7)	30 (76.9)	
** Female**	7 (13.2)	3 (27.3)		1 (6.2)	5 (25.0)		1 (5.3)	9 (23.1)	
** Age (years)**	58.3±9.7	66.0±7.0	0.015	50.1±11.2	54.2±12.2	0.303	58.9±9.3	52.3±10.4	0.022
**Tumor size (mm)**	33.2±16.3	34.8±17.9	0.763	38.3±18.2	38.5±24.9	0.985	43.6±26.4	53.3±32.3	0.258
**Etiology**									
** Non-viral**	5 (9.4)	2 (18.2)		2 (12.5)	5 (25.0)		5 (26.3)	14 (35.9)	
** Viral (HBV, HCV)**	48 (90.6)	9 (81.8)		14 (87.5)	15 (75.0)		14 (73.7)	25 (64.1)	
**Differentiation**			0.517			0.085			0.017
** Well**	23 (43.4)	4 (36.4)		3 (18.8)	0 (0.0)		6 (31.6)	4 (10.3)	
** Moderate**	25 (47.2)	5 (45.5)		11 (68.8)	15 (75.0)		11 (57.9)	22 (56.4)	
** Poor**	5 (9.4)	2 (18.2)		2 (12.5)	5 (25.0)		2 (10.5)	13 (33.3)	
**Vascular invasion**			0.362			0.940			0.619
** Absence**	32 (60.4)	5 (45.5)		7 (43.7)	9 (45.0)		5 (26.3)	8 (20.5)	
** Presence**	21 (39.6)	6 (54.5)		9 (56.3)	11 (55.0)		14 (73.7)	31 (79.5)	
**Multiplicity**			1.000			0.005			1.000
** Single**	44 (83.0)	10 (90.9)		8 (50.0)	19 (95.0)		18 (94.7)	38 (97.4)	
** Multiple**	9 (17.0)	1 (9.1)		8 (50.0)	1 (5.0)		1 (5.3)	1 (2.6)	
**Intrahepatic metastasis**			1.000			0.190			0.733
** Absence**	51 (96.2)	11		14 (87.5)	20		16 (84.2)	30 (76.9)	
** Presence**	2 (3.8)	0		2 (12.5)	0		3 (15.8)	9 (23.1)	
**Preoperative treatment**			1.000			0.202			0.486
** No**	39 (73.6)	8 (72.7)		7 (43.8)	13 (65.0)		13 (68.4)	23 (59.0)	
** Yes**	14 (26.4)	3 (27.3)		9 (56.3)	7 (35.0)		6 (31.6)	16 (41.0)	

HCC, hepatocellular carcinoma; cHC-CC, combined hepatocellular-cholangiocarcinoma

### Survival analysis in EpCAM(−)/K19(−) HCCs, EpCAM(+)/K19(+) HCCs, and cHC-CCs

**Figure 3:**
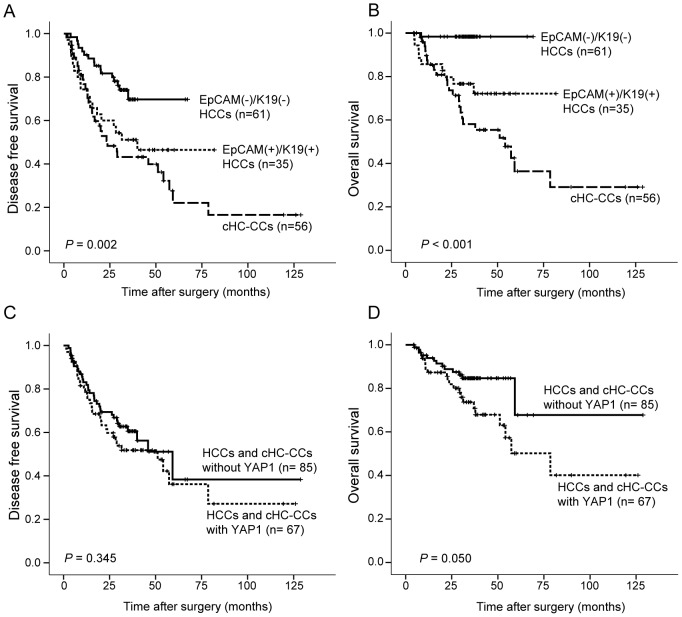
Kaplan–Meier's plot analysis for disease-free survival and overall survival in HCCs and combined hepatocellular-cholangiocarcinomas (cHC-CCs). Kaplan–Meier's plot analysis for disease-free survival (A) and overall survival (B) showing a significant difference among EpCAM(−)/K19(−) HCCs, EpCAM(+)/K19(+) HCCs, and cHC-CCs. Overall survival was relatively worse in HCCs and cHC-CCs with YAP1 expression (D), whereas there was no significant difference in disease-free survival between the two groups.

Overall survival and disease-free survival were evaluated for 152 patients, including 61 cases of EpCAM(−)/K19(−) HCC, 35 cases of EpCAM(+)/K19(+) HCC, and 56 cases of cHC-CC. Six patients who died within one month after an operation were excluded from the survival analysis to avoid any influence of perioperative mortality. The median follow-up time after surgical resection was 32.8 months (4.3–128.7) and 34 patients died of HCC or cHC-CC during follow-up. Disease-free survival showed a statistically significant difference among the three groups: disease-free survival rate was highest for EpCAM(−)/K19(−) HCCs and lowest for cHC-CCs, with EpCAM(+)/K19(+) HCCs falling in between (*P* = 0.002) (Fig. 3A). Overall survival also revealed a statistically significant difference among the three groups (*P*<0.001) (Fig. 3B). Among the patients with cHC-CC, there was no difference between classical type and intermediate-cell subtype patients in overall survival and disease-free survival rate (Figure S1).

When primary liver cancers were divided into two groups according to YAP1 expression, there were 67 cases with YAP1 expression and 85 cases without. Disease-free survival showed no significant difference between these two groups, whereas overall survival rate was relatively lower in primary liver cancers with YAP1 expression compared to those that did not express YAP1 (*P* = 0.050) (Fig. 3C and 3D).

Univariate analysis revealed that larger tumor size (≥4 cm) (*P* = 0.006), history of preoperative treatment (*P*<0.001), vascular invasion (*P*<0.001), intrahepatic metastasis (*P*<0.001), and the histologic groups of cHC-CC and EpCAM(+)/K19(+) HCC (*P* = 0.004) were adverse prognostic factors affecting disease-free survival after resection. In regards to overall survival, larger tumor size (≥4 cm) (*P*<0.001), vascular invasion (*P*<0.001), intrahepatic metastasis (*P*<0.001) and the histologic groups of cHC-CC and EpCAM(+)/K19(+) HCC (*P* = 0.002) were revealed as adverse prognostic factors (Table 4).

**Table 4:** Univariate analysis of disease-free and overall survival rate.

		Disease-free survival			Overall survival		
Variable	N	HR	95% CI	*P*	HR	95% CI	*P*
**Sex**							
** Female**	25	1			1		
** Male**	127	1.021	0.546–1.909	0.946	1.670	0.588–4.745	0.336
**Age (years)**							
** <50**	43	1			1		
** ≥50**	109	1.395	0.791–2.461	0.249	1.344	0.604–2.992	0.469
**Etiology**							
** Viral (HBV, HCV)**	121	1			1		
** Non-viral**	31	1.371	0.790–2.381	0.261	1.307	0.611–2.798	0.490
**Tumor size**							
** <4** **cm**	83	1			1		
** ≥4 cm**	69	1.974	1.213–3.211	0.006	6.925	2.859–16.769	<0.001
**Multiplicity**							
** Single**	134	1.000			1		
** Multiple**	18	1.437	0.731–2.824	0.292	1.251	0.381–4.101	0.712
**Differentiation**							
** Well/moderate**	124	1			1		
** Poor**	28	1.554	0.885–2.726	0.124	1.836	0.867–3.887	0.112
**Preoperative treatment**							
** No**	101	1			1		
** Yes**	51	2.307	1.422–3.744	<0.001	0.978	0.476–2.009	0.951
**Vascular invasion**							
** Absence**	63	1			1		
** Presence**	89	2.841	1.636–4.935	<0.001	14.769	3.534–61.715	<0.001
**Intrahepatic metastasis**							
** Absence**	136	1			1		
** Presence**	16	3.298	1.712–6.350	<0.001	4.563	2.035–10.232	<0.001
**Histologic group**							
** EpCAM(−)/K19(−) HCCs**	61	1		0.004	1		0.002
** EpCAM(+)/K19(+) HCCs**	35	2.148	1.092–4.226	0.026	16.533	2.091–130.707	0.008
** cHC-CCs**	56	2.812	1.530–5.167	0.001	29.442	3.953–219.286	0.001
**YAP1 expression** *							
** Negative**	85	1			1		
** Positive**	67	1.261	0.777–2.046	0.346	1.990	0.988–4.008	0.050

* Nuclear YAP1 expression with moderate to strong intensities in more than 5% of the tumor cells were regarded as positive.

Multivariable analysis indicated that history of preoperative treatment (HR = 2.063, *P* = 0.004) and vascular invasion (HR = 2.240, *P* = 0.007) were independent prognostic factors for disease-free survival after resection. For overall survival, larger tumor size (≥4 cm) (HR = 3.448, *P* = 0.008), vascular invasion (HR = 7.135, *P* = 0.009), and the histologic groups of cHC-CC and EpCAM(+)/K19(+) HCC (*P* = 0.034) were shown to be independent prognostic factors on multivariable analysis (Table 5).

**Table 5:** Independent prognostic factors for disease-free and overall survival by multivariable analysis.

			Disease-free survival			Overall survival		
Variable		N	HR	95% CI	*P*	HR	95% CI	*P*
**Tumor size**								
** <4 cm**		83	1			1		
** ≥4 cm**		69	1.356	0.781–2.354	0.277	3.448	1.378–8.624	0.008
**Preoperative treatment**								
** No**		101	1					
** Yes**		51	2.063	1.258–3.383	0.004			
**Vascular invasion**								
** Absence**		63	1			1		
** Presence**		89	2.240	1.249–4.015	0.007	7.135	1.646–30.927	0.009
**Histologic group**					0.133			0.034
** EpCAM(−)/K19(−) HCCs**		61	1			1		
** EpCAM(+)/K19(+) HCCs**		35	1.922	0.972–3.801	0.060	16.015	1.959–130.96	0.010
** cHC-CCs**		56	1.692	0.896–3.196	0.104	13.887	1.761–109.546	0.013

## Discussion

Among morphologically pure HCCs, cases that express stemness-related markers, such as EpCAM, K19, CD133, etc., have been reported to exhibit more aggressive clinicopathological features, including more frequent vascular invasion, increased angiogenesis, higher recurrence rate, and worse prognosis.[9], [10], [22], [23] In this study, EpCAM(+)/K19(+) HCCs showed poorer histological differentiation, greater vascular invasion, and worse prognosis than EpCAM(−)/K19(−) HCCs.

cHC-CCs are rare primary liver tumors, and can be categorized into classical type cHC-CCs and subtypes with stem cell features.[3] Classical type cHC-CCs contain HCC areas, CC areas and transitional zones, which comprise tumor cells with intermediate morphology resembling stem/progenitor cells. Subtypes with stem cell features include the typical subtype, intermediate-cell subtype, and cholangiocellular subtype, and tumor cells that have phenotypical or immunophenotypical features of stem/progenitor cells are the main component.[3] The gene signatures associated with early liver development and stem cells have been reported to be significantly enriched in cHC-CC.[24] These features suggest that cHC-CC is closely associated with stemness. Moreover, cHC-CCs have been reported to exhibit aggressive characteristics of greater lymph node involvement, vascular invasion, and worse prognosis than HCC.[13], [25]–[27] The present study also revealed that cHC-CCs show more aggressive characteristics of larger tumor size, more frequent vascular invasion and poorer differentiation than EpCAM(−)/K19(−) HCCs. Among these characteristics, larger tumor size and more frequent vascular invasion were also more frequently noted in cHC-CCs than in EpCAM(+)/K19(+) HCCs. Moreover, disease-free survival and overall survival showed a statistically significant difference among cHC-CCs, HCCs with stemness, and HCCs without stemness: disease-free survival rate was highest in EpCAM(−)/K19(−) HCCs and lowest in cHC-CCs, with EpCAM(+)/K19(+) HCCs falling in between.

In a previous study, transgenic mice with liver-specific YAP1 overexpression were reported to develop primary liver tumors, which morphologically resembled human cHC-CCs.[21] In the present study, YAP1 expression was found in 67% of cHC-CCs, 56% of EpCAM(+)/K19(+) HCCs, and 17% of EpCAM(−)/K19(−) HCCs. Such expression was more frequently found in EpCAM(+)/K19(+) HCCs and cHC-CCs than in EpCAM(−)/K19(−) HCCs. In cHC-CCs, YAP1 expression was associated with poorer histological differentiation, and was more frequently noted in transitional zones with features of stem/progenitor cells, compared to HCC areas, although this was not statistically significant.

YAP1 is known to have the ability to induce epithelial mesenchymal transition (EMT), the differentiation of polarized epithelial cells to contractile and motile mesenchymal cells.[28] EMT induction by ectopic expression of either Snail or Twist transcription factors was also reported to lead to cancer stem-cell properties in human breast cancer cells.[29] Interestingly, our previous study revealed that HCCs expressing K19 and/or EpCAM show upregulation of EMT-associated genes and more invasive characteristics than those without.[9] In this study, YAP1 expression was significantly higher in HCCs with stemness than in those without. Taken together, our results suggest that the Hippo-YAP1 pathway might be involved in the pathogenesis of liver cancers with stemness, such as EpCAM(+)/K19(+) HCCs and cHC-CCs, which exhibit aggressive biological behavior. Additionally, YAP1 expression has been reported to be related to poor prognosis in several malignancies, including HCC, non-small cell lung cancer, gastric cancer and colorectal cancer.[30]–[33] In this study, overall survival rate was relatively lower in HCCs and cHC-CCs that expressed YAP1 compared to those that did not, whereas disease free survival showed no difference according to YAP1 expression. Also, YAP1 expression was revealed as a significant prognostic factor affecting overall survival on univariate analysis, but not on multivariate analysis.

In conclusion, this is the first study to provide clinicopathological evidence that YAP1 is more frequently expressed in HCCs expressing stemness-related markers (EpCAM and K19) and in cHC-CCs, compared to HCCs lacking such expression. Our findings suggest that YAP1 expression may contribute to the gain of stemness in HCCs and cHC-CCs, and could be a potential therapeutic target for treatment of these tumors.

## Supporting Information

Figure S1 **Kaplan–Meier's plot analysis for disease-free survival and overall survival in combined hepatocellular-cholangiocarcinomas (cHC-CCs).** There was no difference between classical type and intermediate-cell subtype patients in disease-free survival (A) and overall survival rate (B).
(TIF)

Table S1 Clinicopathological features and YAP1 expression in classical-type and intermediate-cell subtype combined hepatocellular-cholangiocarcinomas
(DOCX)
