# Supplementary figures and images for: Increased Expression of Yes-Associated Protein 1 in Hepatocellular Carcinoma with Stemness and Combined Hepatocellular-Cholangiocarcinoma
Source: PLoS One. 2013 Sep 24;8(9):e75449. doi: 10.1371/journal.pone.0075449 (PMC3782432; doi:10.1371/journal.pone.0075449)

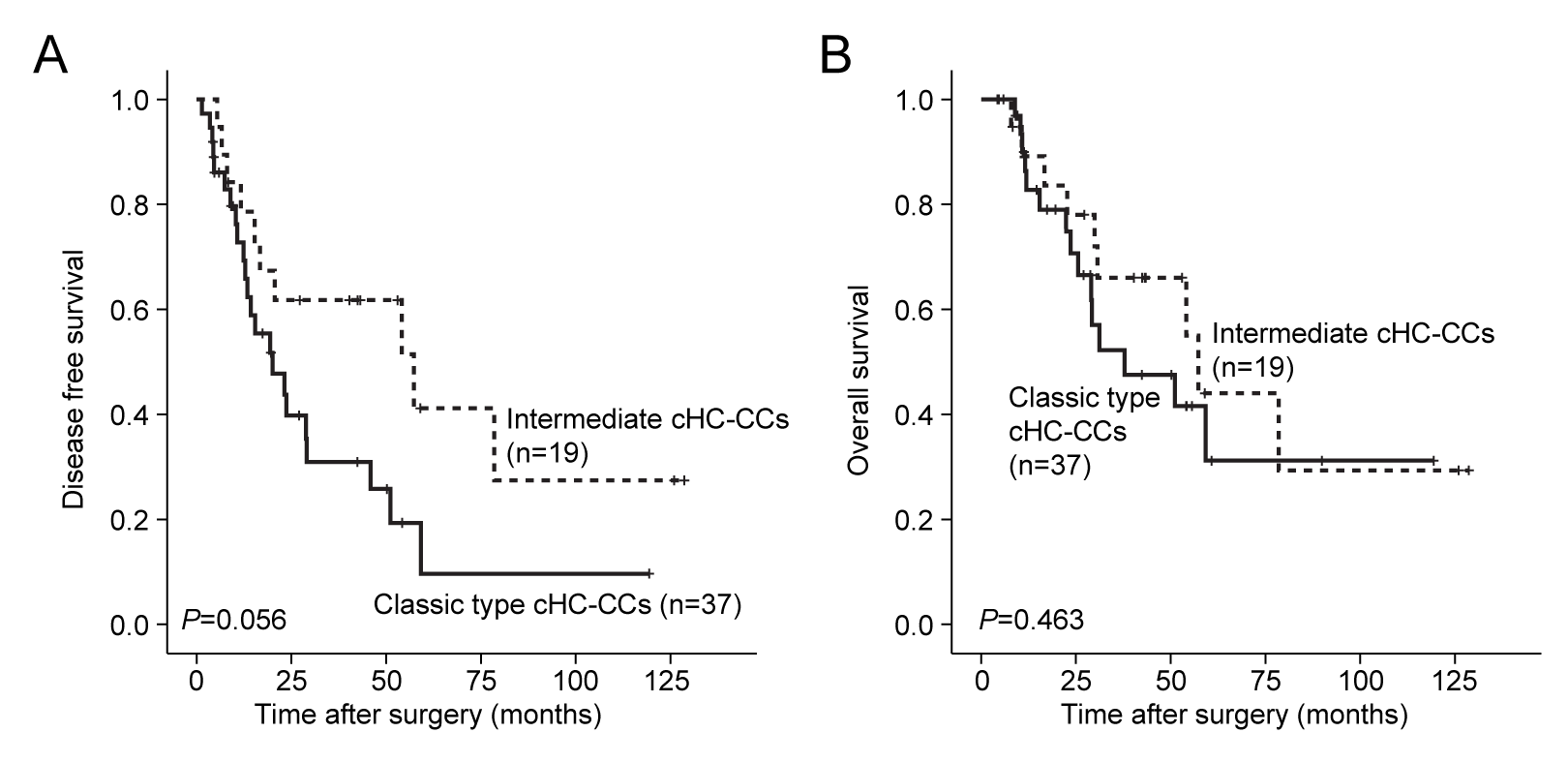

Supplement: Figure S1 — Kaplan–Meier's plot analysis for disease-free survival and overall survival in combined hepatocellular-cholangiocarcinomas (cHC-CCs). There was no difference between classical type and intermediate-cell subtype patients in disease-free survival (A) and overall survival rate (B). (TIF) [file pone.0075449.s001.tif]
