# Supplementary material for: Increased Expression of Yes-Associated Protein 1 in Hepatocellular Carcinoma with Stemness and Combined Hepatocellular-Cholangiocarcinoma
Source: PLoS One. 2013 Sep 24;8(9):e75449. doi: 10.1371/journal.pone.0075449 (PMC3782432; doi:10.1371/journal.pone.0075449)
Supplement: Table S1 — Clinicopathological features and YAP1 expression in classical-type and intermediate-cell subtype combined hepatocellular-cholangiocarcinomas (DOCX) [file pone.0075449.s002.docx]

**Table S1.** Clinicopathological features and YAP1 expression in classical-type and intermediate-cell subtype combined hepatocellular-cholangiocarcinomas

|  | **Classical-type (n=38) (%)** | **Intermediate-cell subtype (n=20) (%)** | ***P*** |
| --- | --- | --- | --- |
| **Sex** |  |  | 0.724 |
| **Male** | 32 (84.2) | 16 (80.0) |  |
| **Female** | 6 (15.8) | 4 (20.0) |  |
| **Age (years)** | 56.3$\pm$9.0 | 50.8$\pm$12.3 | 0.055 |
| **Etiology** |  |  | 0.001 |
| **Non-viral** | 7 (18.4) | 12 (60.0) |  |
| **HBV** | 27 (71.1) | 8 (40.0) |  |
| **HCV** | 4 (10.5) | 0 (0.0) |  |
| **Tumor size (mm)** | 48.7$\pm23$.6 | $52.9\pm$41.4 | 0.677 |
| **Differentiation** |  |  | 0.463 |
| **Well** | 6 (15.8) | 4 (20.0) |  |
| **Moderate** | 21 (55.3) | 12 (60.0) |  |
| **Poor** | 11 (28.9) | 4 (20.0) |  |
| **Vascular invasion** |  |  | 0.095 |
| **Absence** | 6 (15.8) | 7 (35.0) |  |
| **Presence** | 32 (84.2) | 13 (65.0) |  |
| **Multiplicity** |  |  | 1.000 |
| **Single** | 37 (97.4) | 19 (95.0) |  |
| **Multiple** | 1 (2.6) | 1 (5.0) |  |
| **Intrahepatic metastasis** |  |  | 0.515 |
| **Absence** | 29 (76.3) | 17 (85.0) |  |
| **Presence** | 9 (23.7) | 3 (15.0) |  |
| **Preoperative treatment** |  |  | 0.051 |
| **No** | 20 (52.6) | 16 (80.0) |  |
| **Yes** | 18 (47.4) | 4 (20.0) |  |
| **YAP1 expression*** |  |  | 0.361 |
| **Negative** | 14 (36.8) | 5 (25.0) |  |
| **Positive** | 24 (63.2) | 15 (75.0) |  |

*Nuclear YAP1 expression with moderate to strong intensities in more than 5% of the tumor cells were regarded as positive.
